# Feature and decision-level fusion for schizophrenia detection based on resting-state fMRI data

**DOI:** 10.1371/journal.pone.0265300

**Published:** 2022-05-24

**Authors:** Ali H. Algumaei, Rami F. Algunaid, Muhammad A. Rushdi, Inas A. Yassine

**Affiliations:** Department of Biomedical Engineering and Systems, Faculty of Engineering, Cairo University, Giza, Egypt; University of California Los Angeles, UNITED STATES

## Abstract

Mental disorders, especially schizophrenia, still pose a great challenge for diagnosis in early stages. Recently, computer-aided diagnosis techniques based on resting-state functional magnetic resonance imaging (Rs-fMRI) have been developed to tackle this challenge. In this work, we investigate different decision-level and feature-level fusion schemes for discriminating between schizophrenic and normal subjects. Four types of fMRI features are investigated, namely the regional homogeneity, voxel-mirrored homotopic connectivity, fractional amplitude of low-frequency fluctuations and amplitude of low-frequency fluctuations. Data denoising and preprocessing were first applied, followed by the feature extraction module. Four different feature selection algorithms were applied, and the best discriminative features were selected using the algorithm of feature selection via concave minimization (FSV). Support vector machine classifiers were trained and tested on the COBRE dataset formed of 70 schizophrenic subjects and 70 healthy subjects. The decision-level fusion method outperformed the single-feature-type approaches and achieved a 97.85% accuracy, a 98.33% sensitivity, a 96.83% specificity. Moreover, feature-fusion scheme resulted in a 98.57% accuracy, a 99.71% sensitivity, a 97.66% specificity, and an area under the ROC curve of 0.9984. In general, decision-level and feature-level fusion schemes boosted the performance of schizophrenia detectors based on fMRI features.

## 1 Introduction

Early diagnosis of mental disorders is considered a challenging task. Schizophrenia is one of these chronic mental disorders that typically appear in late adolescence or early adulthood and affect about 1% of the population around the world [1–4]. This disorder is characterized by hallucinations, delusions and negative symptoms such as social withdrawal, self neglect, etc. [5].

Within the last decade, most studies have shown that machine learning and pattern classification techniques are useful for finding potential biomarkers for schizophrenia based on different types of discriminative features: structural features [6–18], functional features [19–25] or combination of functional and structural features [12, 26–27].

Structural studies reveal the anatomical changes of the brain such as expansion of the lateral ventricles [28], decreased volumes of medial temporal structures such as the amygdala, the parahippocampal gyrus, the hippocampus [29–31], the prefrontal cortex [32, 33], the superior temporal gyrus [30], and the inferior parietal lobule [34, 35]. Furthermore, the abnormal enlargement of the left hemisphere compared to the right hemisphere can be considered as a sign of schizophrenia in male patients [36, 37]. Moreover, other studies revealed structural correlations between temporal lobe and prefrontal brain volumes [38, 39], in addition to some disturbances of functional brain connectivity between temporal and frontal lobes. These changes confirm the disconnectivity hypothesis [40, 41], which explains the widespread problems of the functional connectivity in schizophrenia.

Resting-state functional magnetic resonance imaging (Rs-fMRI) is considered as one of the most promising tools in the diagnosis of different mental disorders specially schizophrenia, as it allows exploring the brain functions at rest [24, 42]. Recently, most studies have examined the automatic diagnosis of brain disorders using Rs-fMRI [43–51].

Slow activity fluctuations, measured by the blood oxygenation level dependent (BOLD) signal in Rs-fMRI, allow defining the resting-state networks and obtaining the correlated activity between the various brain regions. The functional connectivity [52, 53] maps can be created through calculating the correlation measures of these fluctuations. These maps may be used as biomarkers or distinctive features for mental or individual variations. Tang *et al*. [54] reached an accuracy of 93.2% using 22 schizophrenic patients and 22 healthy subjects based on functional connectivity. Yu *et al*. [55] achieved a 62% accuracy for a system trained and tested on the functional connectivity measures of 24 schizophrenic patients, 25 healthy siblings and 22 healthy subjects. The major drawback of the previously stated studies is the employment of the small datasets. Guo *et al*. [56] achieved an accuracy of 75% using a dataset formed of 46 unaffected siblings of schizophrenic patients and 50 healthy subjects based only on the fractional amplitude of low frequency fluctuations (fALFF). While this study enjoys a relatively large dataset, it still suffers from the limited investigation of suitable features for schizophrenia diagnosis and the relatively low reported accuracy. Chyzhyk *et al*. [57] employed a classification system based on an Rs-fMRI dataset provided by the Center of Biomedical Research Excellence (COBRE) in mental illness and brain function. Experiments were conducted with four feature types, namely: regional homogeneity (ReHo), voxel-mirrored homotopic connectivity (VMHC), fractional amplitude of low-frequency fluctuations (fALFF) and amplitude of low-frequency fluctuations (ALFF), where the reported accuracy reached 91% using the VMHC features.

Recently, data fusion techniques have been explored for improving the performance of detection and classification systems in numerous applications [58, 59]. Fusion can be generally performed on different levels, especially the feature and decision levels. Such fusion schemes have been particularly applied in biomedical signal analysis [60], and multiple medical imaging modalities [61]. In particular, fusion of brain imaging data has been consistently improving the performance of automated systems for mental illness detection [62].

**Fig 1:**
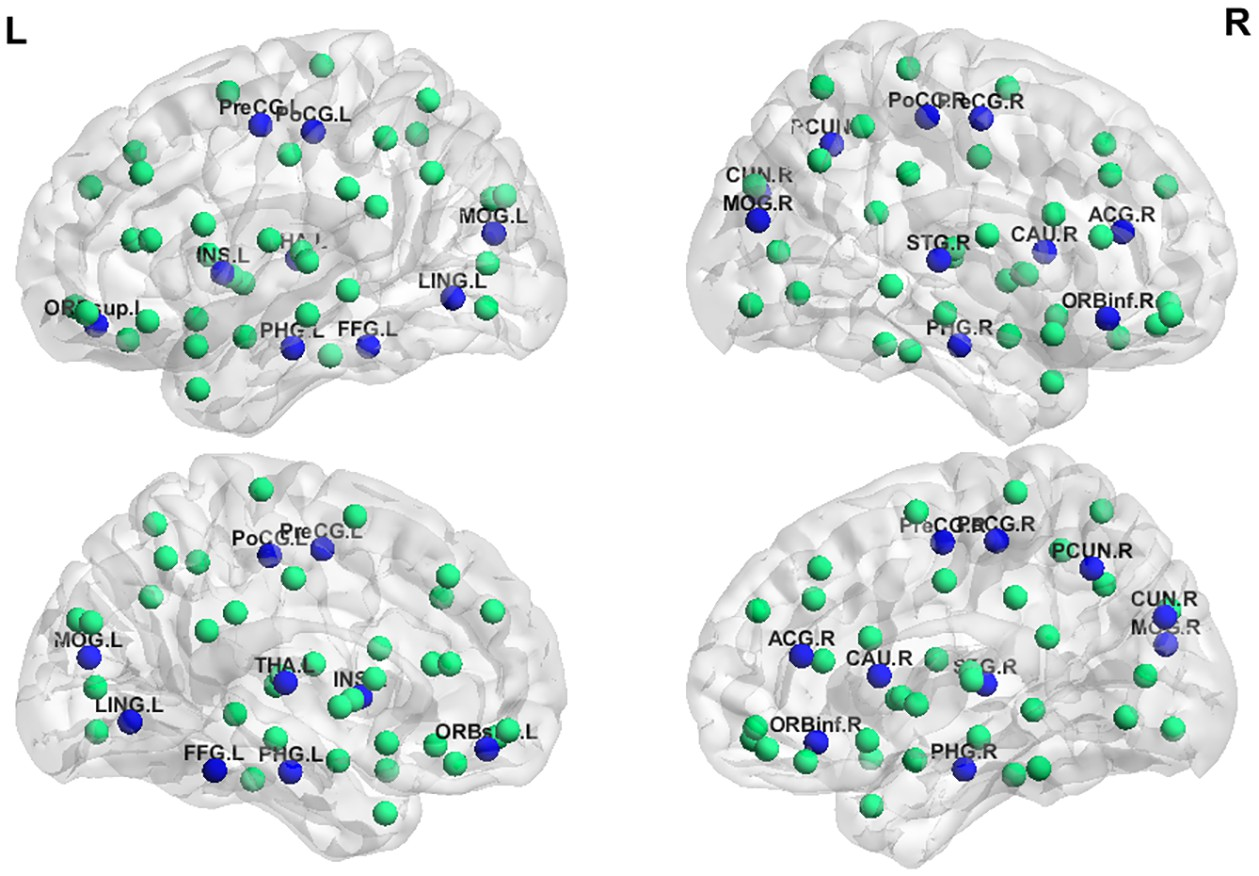
A general block diagram of the proposed schizophrenia detection schemes. The single-feature-type schemes follow the red arrows. The feature-fusion schemes are indicated by the blue arrows. For the decision-level fusion schemes, the orange arrows trace the path from the single-feature-type schemes to the final fused decisions.

In this paper, we investigated a schizophrenia detection framework based on the individual ReHo, VMHC, fALFF and ALFF feature types. The resultant feature sets were optimized using different feature selection techniques. For this framework, we explored decision-level fusion between the outputs of classifiers trained on single-feature types. We also investigated feature-level fusion schemes in which pairs, triples, and quadruples of feature types are combined and used for classifier training. In the first single-feature-type scheme, each of the four feature types is exploited independently. The second scheme applies majority-vote fusion on the decision level to three classifiers trained on the ReHo, VMHC, and fALFF features, respectively. The third scheme explores feature-level fusion through combining different types of features before feature selection. The different schemes are shown in Fig 1, where the colored arrows represent the flow between the different modules forming the different schemes. The red arrows trace the flow of the single-feature-type schemes, while the blue arrows are associated with the feature-level fusion schemes. For the decision-level fusion schemes, the single-feature-type schemes are combined through the orange arrows to reach the final decisions.

The rest of the paper is organized as follows. Section II describes the whole system modules including dataset description, preprocessing, resting-state functional activity measures, feature selection, and pattern classification. Section III reports and discusses the experimental results. Section IV concludes the paper and gives suggestions for future work.

## 2 Materials and methods

### 2.1 Dataset description

We employed a dataset created for studying the neural mechanisms of schizophrenia. This dataset was collected by the Center for Biomedical Research Excellence (COBRE) http://fcon1000.projects.nitrc.org/indi/retro/cobre.html through the Mind Research Network for Neurodiagnostic Discovery (MRN) at the University of New Mexico. The dataset was collected in accordance with the recommendations of the Declaration of Helsinki. The COBRE data acquisition protocol was approved by the Institutional Review Boards of all of the participating institutions. Informed written consent was obtained from all participants at each site. The dataset includes raw functional and anatomical MR data for 140 subjects distributed equally for normal and schizophrenic patients. Diagnostic information was collected using the structured clinical interview utilized for DSM disorders (SCID). A multi-echo MPRAGE (MEMPR) sequence was utilized for anatomical imaging with the following parameters: TR/TE/TI = 2530/[1.64, 3.5, 5.36, 7.22, 9.08]/900 ms, flip angle = 7, slab thickness = 176 mm, FOV = 256 × 256 mm, data matrix = 256 × 256 × 176, number of echoes = 5, total scan time = 6 min, voxel size = 1 × 1 × 1 mm, pixel bandwidth = 650 Hz. With 5 echoes, the TI, TR and time to encrypt partitions for MEMPR are similar to those of conventional MPRAGE, and lead to similar GM/WM/CSF contrast. Data for Rs-fMRI was collected using echo planar imaging (EPI) with ramp sampling correction using the intercomissural line (AC-PC) as a reference (TR: 2 s, TE: 29 ms, matrix size: 64 × 64, 32 slices, voxel size: 3 × 3 × 4*mm*^3^). Rs-fMRI, anatomical MRI, and phenotypic data are recorded for every subject. A brief summary of the demographic data found in the COBRE schizophrenia dataset is shown in Table 1.

**Table 1:** Summary of participant demographics in the COBRE schizophrenia dataset.

	Patients	Controls
No. of subjects	70	70
Age	37.9 (18-65)	35.8 (18-65)
Gender (M/F)	56/14	48/22
Handedness (R/L)	59/11	67/3

### 2.2 Data preprocessing

All the preprocessing steps were executed in MATLAB using the data processing & analysis for brain imaging (DPABI) software tool [63]. For each participant, slice time correction was applied for interleaved acquisition. Head motion correction based on Friston’s 24-parameter motion model [64] was performed. Co-registration of structural and functional images in order to map the functional information to the anatomical space was executed. Then, the images were spatially normalized to the Montreal Neurological Institute (MNI) standard using the DARTEL template [65] and resampled to 3 × 3 × 3 *mm*^3^. The generated images were spatially smoothed with a 4-mm full-width half-maximum (FWHM) Gaussian kernel. Moreover, the images were linearly detrended and temporally filtered by a bandpass filter (0.01–0.1 Hz) to reduce low-frequency drifts and remove physiological high-frequency noise [66].

### 2.3 Resting-state functional activity measures

We explain here the functional activity measures calculated over the Rs-fMRI dataset.

#### 2.3.1 ALFF and fALFF features

The ALFF [67] and fALFF [68] features measure the magnitude of low frequency fluctuations (LFFs) of the BOLD signal. The ALFF features are calculated as the average power spectrum, obtained across the range (0.01—0.1 Hz) for each voxel. The fALFF features represent the ratio of the signal power of the low-frequency range (0.01 to 0.1 Hz) to the power associated with the total detectable frequency range (0 to 0.25 Hz) [67].

#### 2.3.2 VMHC features

This feature set measures the brain functional homotopy through a voxel-wise measure of connectivity among the brain hemispheres, under the assumption of synchrony in spontaneous brain activity among the homotopic regions for each hemisphere. An approximation of the homotopic connectivity is computed between an individual voxel in one of the brain hemispheres and its symmetric counterpart in the other hemisphere of the brain, assuming morphology regularity between them. This connectivity is calculated based on the Pearson correlation coefficient between voxel pairs across the hemispheres. Then, the Pearson correlation coefficient value is converted into a Fisher z-transformed value representing the VMHC activity measures [69].

#### 2.3.3 ReHo features

This feature set measures the similarity between the time series of a particular voxel and those of its nearest neighbors [70]. To compute the similarities inside a voxel cluster, Kendall’s coefficient of concordance (KCC) is applied. Here, each cluster contains 27 neighboring voxels. For each subject, a voxel-based map is built by standardizing and smoothing the KCC values based on a 4-mm FWHM Gaussian kernel [71].

For each of the above-mentioned four feature types, 271633 features were originally extracted.

### 2.4 Feature selection

To improve the classification performance, we examined 15 different feature selection measures [72]. Each feature selection algorithm provides a list of features ranked by the feature strength or discriminability from the most discriminating feature to the least discriminating one. Then, we followed a sequential forward selection approach. That is, we use the most significant feature, followed by the most two significant features, where the system has been trained and the performance is evaluated using the validation dataset for each case. This process is continued where the significant feature subset is enlarged by adding one feature at a time. This process was employed for the different feature selection algorithms. We selected the following four top-performing feature selection algorithms: feature selection via concave minimization (FSV) [73], L0-norm [74], Relief [75] and Wilcoxon sum-rank test [76].

### 2.5 Decision and feature fusion

Several schemes for decision-level and feature-level fusion are investigated to improve the schizophrenia detection performance. For decision-level fusion, we applied majority voting on three SVM classifiers which are based on the ReHo, VMHC, and fALFF feature types, respectively. Feature-level fusion combines different fMRI feature types to exploit the strengths of each type.

### 2.6 Support vector machine (SVM) classification

A support vector machine (SVM) is a linear classifier that learns the best hyperplane that has the maximum possible distance to the closest data point in the training set belonging to any class using the support vectors. Thus, a SVM is typically more robust compared to other classifiers. The SVM effectiveness is enhanced mainly through the employment of the kernel trick in order to handle data nonlinearity in the feature space. This trick basically transforms the data points into a higher-dimensional feature space in order to increase the linear separability between the data points. The most commonly used kernels include linear, quadratic, and radial basis function (RBF) kernels [77].

After feature selection and fusion, supervised machine learning procedures were used to discriminate the schizophrenic patients from healthy subjects. The COBRE dataset, employed in this work, was divided into training, validation and testing subsets. Training and testing of the classifiers were performed using the LIBSVM http://www.csie.ntu.edu.tw/cjlin/libsvm/ library. SVM, with a linear kernel, was employed for the classification task for all proposed schemes, with soft margin C = 10. Five repetitions of nested loop 10-fold cross-validation were performed, in this study, in order to demonstrate the robustness of the system, where the inner 10-fold cross validation was employed only in the feature selection task, i.e. selecting the optimum number of features. It is worth noting that the inner 10-fold cross-validation was employed only in selecting the optimum number of features, while the SVM kernel and parameters were selected by trial and error.

For each fold in the outer loop, one-tenth of the data is randomly selected for the testing and performance evaluation, while the rest is employed in training and validation in the inner 10-fold cross-validation loop. In this inner loop, one-tenth of the remaining samples is randomly selected for the validation and optimization of the feature selection process, while the rest is employed for classifier training. Accuracy, specificity and sensitivity are used to evaluate the classifier performance, select the hyper parameters and verify the system robustness. The overall system performance was evaluated using the accuracy, sensitivity and specificity, which are computed as follows:

$$\begin{array}{c} Accuracy=\frac{TP+TN}{N} \end{array}$$ (1)

$$\begin{array}{c} Specificity=\frac{TN}{TN+FP} \end{array}$$ (2)

$$\begin{array}{c} Sensitivity=\frac{TP}{TP+FN} \end{array}$$ (3)

where the true positive (TP) is the number of correctly classified schizophrenia patients, the false positive (FP) is the number of healthy subjects incorrectly classified as schizophrenia patients, the true negative (TN) is the number of correctly classified healthy subjects, and the false negative (FN) is the number of schizophrenia patients incorrectly classified as healthy subjects. Moreover, the 5x2 cross-validation statistical test was employed to measure the statistical significance of the difference in accuracy between the classifier based on the ReHo activity measure and the classifiers based on other activity measures [78]. The test is carried out as follows. Let A be the ReHo-based classifier and B be the classifier based on another activity measure. The null hypothesis is that the ReHo-based classifier A has the same accuracy as the other classifier B. The alternative hypothesis is that the two classifiers have different accuracies. For each classifier, five repetitions of 2-fold cross-validation are made. Then, for each pair of classifiers, the differences in accuracy are used to compute the following t-statistic:

$$\begin{array}{c} t=\frac{p_{1}^{\left(1\right)}}{\sqrt{\frac{1}{5}\sum_{i=1}^{5}S_{i}^{2}}} \end{array}$$ (4)

where

-
$p_{1}^{\left(1\right)}$ is the difference of the classifier scores for the first fold of the first iteration,
-
$S_{i}^{2}$ is the estimated variance of the score difference for the *i*^*th*^ iteration (This variance is computed as $(p_{i}^{\left(1\right)}-{\bar{p}}_{i}{)}^{2}+(p_{i}^{\left(2\right)}-{\bar{p}}_{i}{)}^{2}$),
-
$p_{i}^{\left(j\right)}$ is the difference of the classifier scores for the *j*^*th*^ fold of the *i*^*th*^ iteration,
-
${\bar{p}}_{i}=\frac{\left(p_{i}^{\left(1\right)}+p_{i}^{\left(2\right)}\right)}{2}$ is the mean score for the *i*^*th*^ repetition over the two associated folds.

The t-statistic is assumed to follow a t-distribution with 5 degrees of freedom. We assume a significance level of 0.05. The corresponding threshold is t* = 2.57. For any two classifiers, the null hypothesis is rejected (i.e. the difference in accuracy for the two classifiers is statistically significance) if |*t*| > t*. Thus, an absolute t-statistic larger than t* indicates that the null hypothesis can be rejected and hence that the ReHo-based classifier accuracy is indeed different from the accuracy of the other classifier.

## 3 Results

### 3.1 Classification outcomes

We experimented with 15 feature selection algorithms and reported the top four best performing ones as shown in Table 2.

**Table 2:** Classification accuracy (%) with feature selection algorithms and feature types.

Algorithms / Feature type	ALFF	fALFF	VMHC	ReHo
**FSV**	**93.57**	**95.71**	**95.00**	**96.42**
L_0_	92.85	92.56	93.57	95.71
Relief	80.14	81.42	80.00	82.14
Wilcoxon	81.42	82.14	83.57	82.14

Our experimental results show that the FSV method gives the best performance for the COBRE http://fcon1000.projects.nitrc.org/indi/retro/cobre.html dataset. The FSV algorithm lists the features according to their discriminability. In our experiments, we used the best single feature to train and test a SVM classifier and obtain the corresponding average validation accuracy over 10 folds. Then, we used the best two features to train and test a SVM classifier and obtain the corresponding average validation accuracy. At each stage of the experiments, the number of used features was increased until all of the features were used for training and validation. The best number of features corresponding to the classifier with the highest average validation accuracy was selected and employed for testing in the outer loop.

We found 95% confidence intervals for the classifier performance using the Wilson score interval method [79, 80]. Among the four types of activity measures (i.e. ALFF, fALFF, ReHo and VMHC), the best average test accuracy of 94.57% is achieved by using the ReHO activity measure with 83 discriminative features as shown in Table 3.

**Table 3:** Schizophrenia detection results with single-feature-type classifiers and decision-level classifiers. Feature selection was applied to each of the four single feature types (ALFF, fALFF, ReHo and VMHC). The decision-level classifier used all feature types except for the ALFF one, which has the worst performance. For comparison, the last column shows the corresponding accuracies obtained by Chyzhyk et al. [57].

Type of Features	# Features	Sensitivity (%)	Specificity (%)	Accuracy (%)	Accuracy (%) in [57]
ALFF	84	81.33±0.04	**99.50± 0.008**	90.71±0.03	87.67
fALFF	73	91.56±0.03	93.36±0.028	92.71±0.03	82.19
VMHC	75	**94.25± 0.027**	93.89±0.027	93.85±0.027	84.19
ReHo	**83**	94.08±0.027	95.78±0.023	**94.57±0.027**	91.19
Decision-fusion classifier (fALFF, ReHo and VMHC)	N.A	**98.33±0.06**	**96.83± 0.10**	**97.85 ±0.09**	N.A

**Fig 2:**
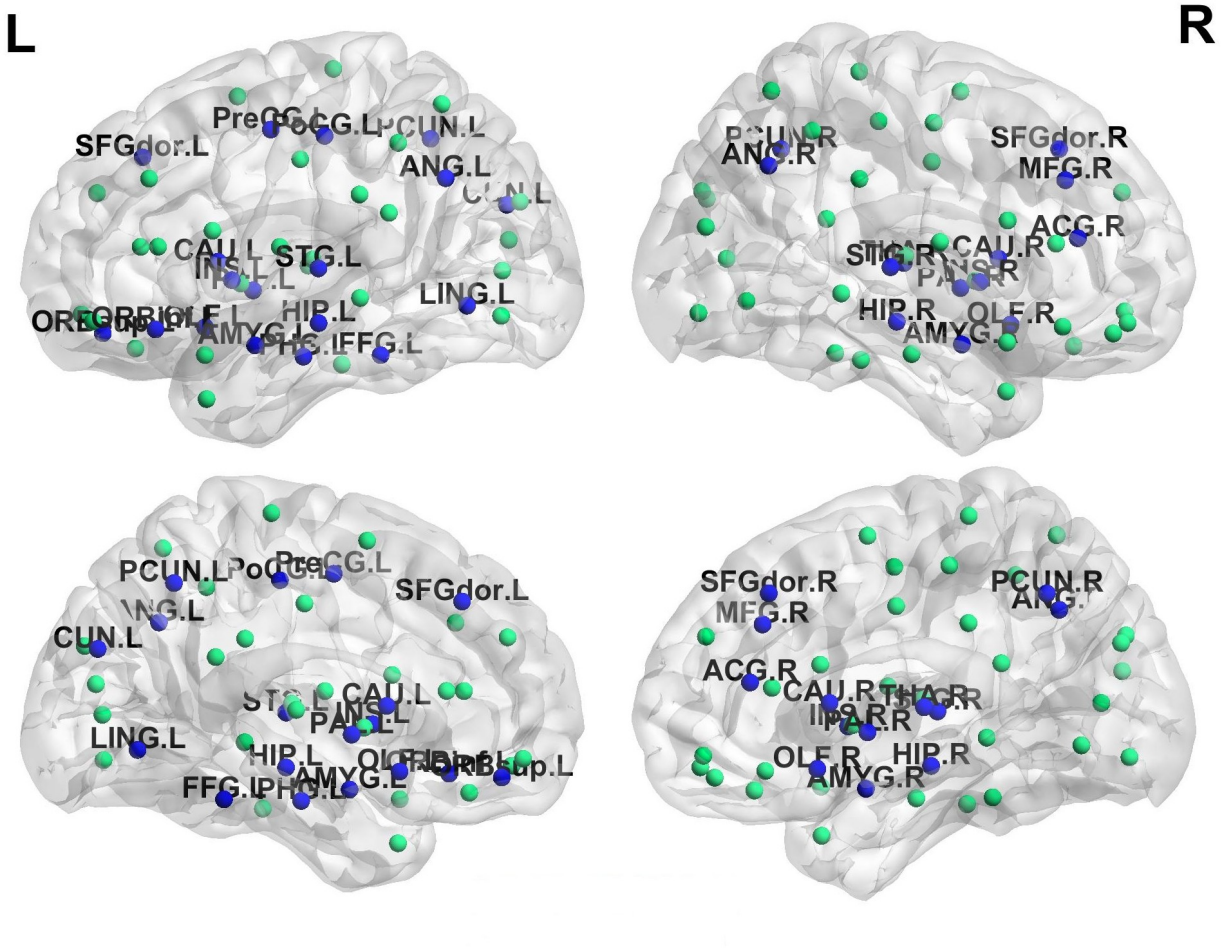
Average accuracy calculation for the decision-level fusion scheme.

We investigated the fusion of decisions and features based on Rs-fMRI activity in order to improve the classification performance. The decision-fusion scheme, shown in Fig 2, computes the ReHo, VMHC, and fALFF features, classifies each feature type using a SVM, and fuses the three decisions. Then, a majority vote is carried among the three classifiers to get the final decision. Table 3 shows the results of the decision-level fusion scheme of Fig 2. Table 3 shows the sensitivity, specificity, and accuracy measures on the test set for each feature type. Our accuracy based on fALFF (92.71%), in Table 3, is significantly better than the 75% accuracy reported by Guo et al. [56]. Moreover, we achieved better results than those obtained by Chyzhyk et al. [57] based on the 4 features types as shown in Table 3. Furthermore, we investigated feature-level fusion schemes. Table 4 shows schizophrenia detection results with different pairwise, triple, and quadruple combinations of the ALFF, fALFF, ReHo and VMHC feature types. Feature selection was applied after combining the features, and the number of features associated with the minimum validation error was selected for each combination. The pairwise combination of the ALFF and fALFF feature types resulted in the best accuracy (97.71%), specificity (97.80%), and sensitivity 98.80%. The ALFF, fALFF, and VMHC combination achieves the best performance among all triple combinations. As well, feature-level fusion of the four feature types leads clearly to the best overall performance metrics with an accuracy of 98.71% and a sensitivity of 99.71%.

**Table 4:** Schizophrenia detection results with feature-level fusion of different pairwise, triple, and quadruple combinations of single feature types.

	Type of Features	# Features	Sensitivity (%)	Specificity (%)	Accuracy (%)
Pairwise features	ALFF+fALFF	91	**98.80± 0.12**	97.80±0.0.017	**97.71±0.017**
	ALFF+ReHo	72	97.40±0.018	97.64±0.017	97.42±0.018
	ALFF+VMHC	71	96.93±0.020	96.86±0.020	96.71±0.021
	fALFF+ReHo	73	95.08±0.025	98.51±0.014	97.42±0.018
	fALFF+VMHC	84	96.85±0.020	98.00±0.016	97.57±0.017
	ReHo+VMHC	75	95.61±0.023	**99.77±0.005**	97.57±0.017
Triple features	ALFF+fALFF+ReHo	82	**98.62± 0.013**	98.62±0.013	97.85±0.016
	ALFF+fALFF+VMHC	70	97.21±0.019	**98.72±0.013**	**97.85±0.016**
	ALFF+ReHo+VMHC	77	92.46±0.030	95.26±0.024	93.85±0.027
	fALFF+ReHo+VMHC	85	95.22±0.024	97.35±0.018	95.71±0.023
Quadruple features	ALFF+fALFF+ReHo+VMHC	**95**	**99.71±0.006**	**97.66±0.016**	**98.71±0.013**

### 3.2 Statistical significance testing

In order to test the statistical significance of the performance differences between the classifiers listed in Tables 3 and 4, we computed t-statistics based on Eq (4). The classifier based on the ReHo activity measure was employed as the reference algorithm since it provided the highest accuracy in the case of single-feature-type classifiers. The resulting p-values for all significance tests associated with single-feature-type classifiers and the decision-level classifier are listed in Table 5. On the one hand, the statistical results show that the difference in accuracy between the ReHo-based and VMHC-based classifiers is not statistically significant. On the other hand, the differences in accuracy between the ReHo-based classifier and the fALFF-based classifier, the ALFF-based classifier, and the decision-level fusion classifier are statistically significant. For the feature fusion classifiers, the p-values listed in Table 6 show statistically significant improvements for all classifiers (except for two classifiers: the ALFF-ReHo-VMHC and fALFF-ReHo-VMHC classifiers) over the ReHo-based classifier.

**Table 5:** P-values for test of significance of the outcomes of the classifiers based on single-feature-type classifiers and decision-level classifier. Statistical comparison is made with respect to the ReHo-based classifier.

Type of Features	Sensitivity	Specificity	Accuracy
ALFF	0.01	0.001	0.003
fALFF	0.003	0.005	0.006
VMHC	0.06	0.10	0.08
ReHo	Ref	Ref	Ref
Decision-fusion classifier (fALFF, ReHo and VMHC)	0.02	0.005	0.006

**Table 6:** P-values for test of significance of the outcomes of the classifiers based on feature-level fusion of different pairwise, triple, and quadruple combinations of single feature types. Statistical comparison is made with respect to the ReHo-based classifier.

	Type of Features	Sensitivity	Specificity	Accuracy
Pairwise features	ALFF+fALFF	0.001	0.004	0.003
	ALFF+ReHo	0.004	0.006	0.003
	ALFF+VMHC	0.02	0.04	0.012
	fALFF+ReHo	0.004	0.005	0.003
	fALFF+VMHC	0.001	0.001	0.002
	ReHo+VMHC	0.005	0.003	0.002
Triple features	ALFF+fALFF+ReHo	0.006	0.004	0.008
	ALFF+fALFF+VMHC	0.007	0.006	0.008
	ALFF+ReHo+VMHC	0.10	0.07	0.08
	fALFF+ReHo+VMHC	0.02	0.03	0.14
Quadruple features	ALFF+fALFF+ReHo+VMHC	0.005	0.006	0.001

### 3.3 ROC analysis

Receiver operating characteristic (ROC) curves were generated for each of the 10 folds of each experiment. An average ROC curve can be obtained by projecting curves from two-dimensional space onto a single dimension and averaging them traditionally. However, this projection raises questions of appropriateness and conservation of the characteristics of interest. The vertical averaging [81] is employed to plot the ROC curvse, where the FP rates are fixed and the corresponding TP are averaged.

**Fig 3:**
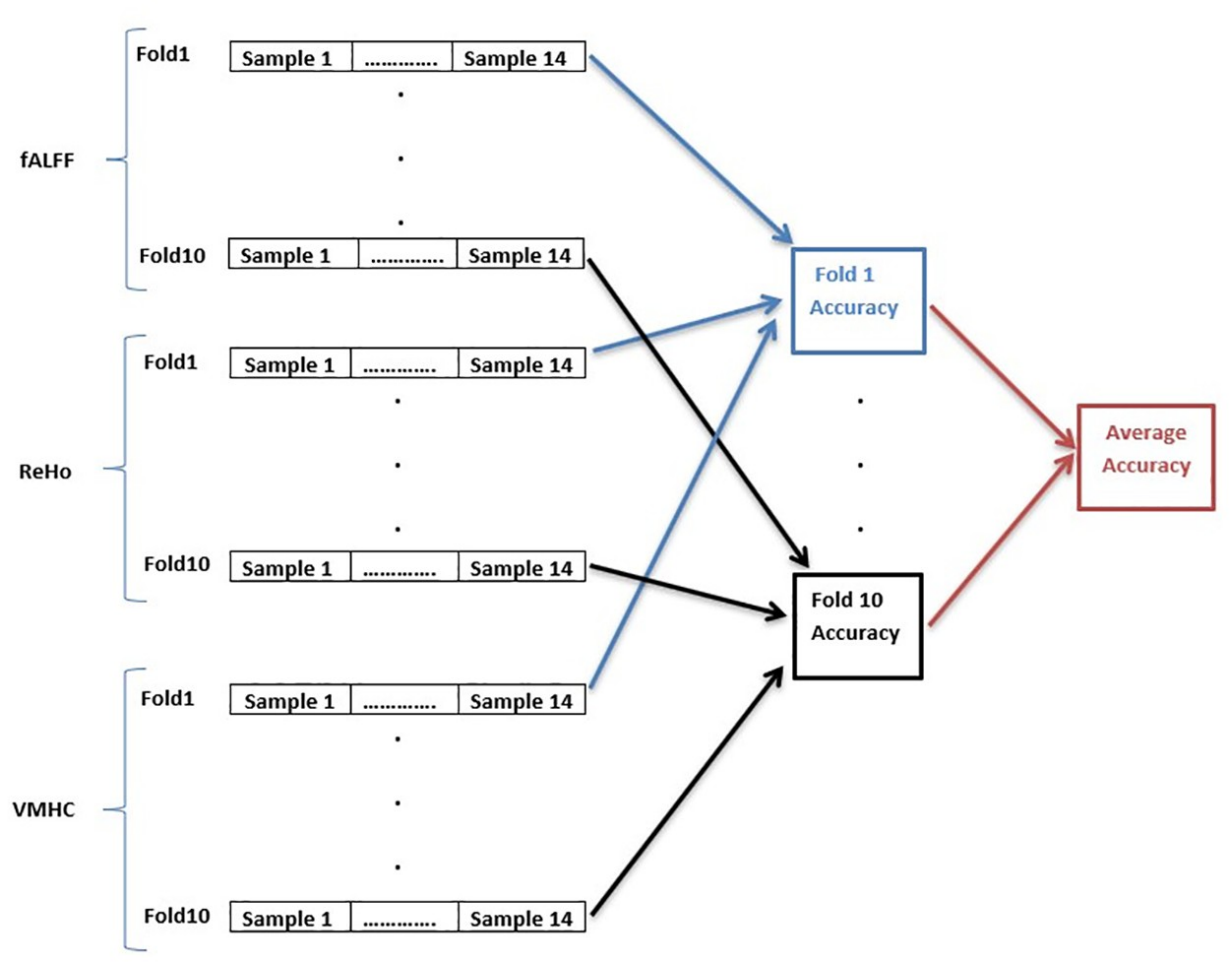
ROC curves for schizophrenia classification using different feature combinations and ROC vertical averaging where the numbers in brackets denote the area under the curve (AUC): (a) Single feature types. (b) Pairwise feature type combinations. (c) Triple and quadruple combinations of features.

We reported the results of vertical averaging method which achieves the best performance in comparison with the threshold averaging method. Fig 3(a)–3(c) shows the results based on vertical averaging for single, pairwise, triple and quadruple combinations of features, respectively.

### 3.4 Discriminative feature mapping

**Fig 4:**
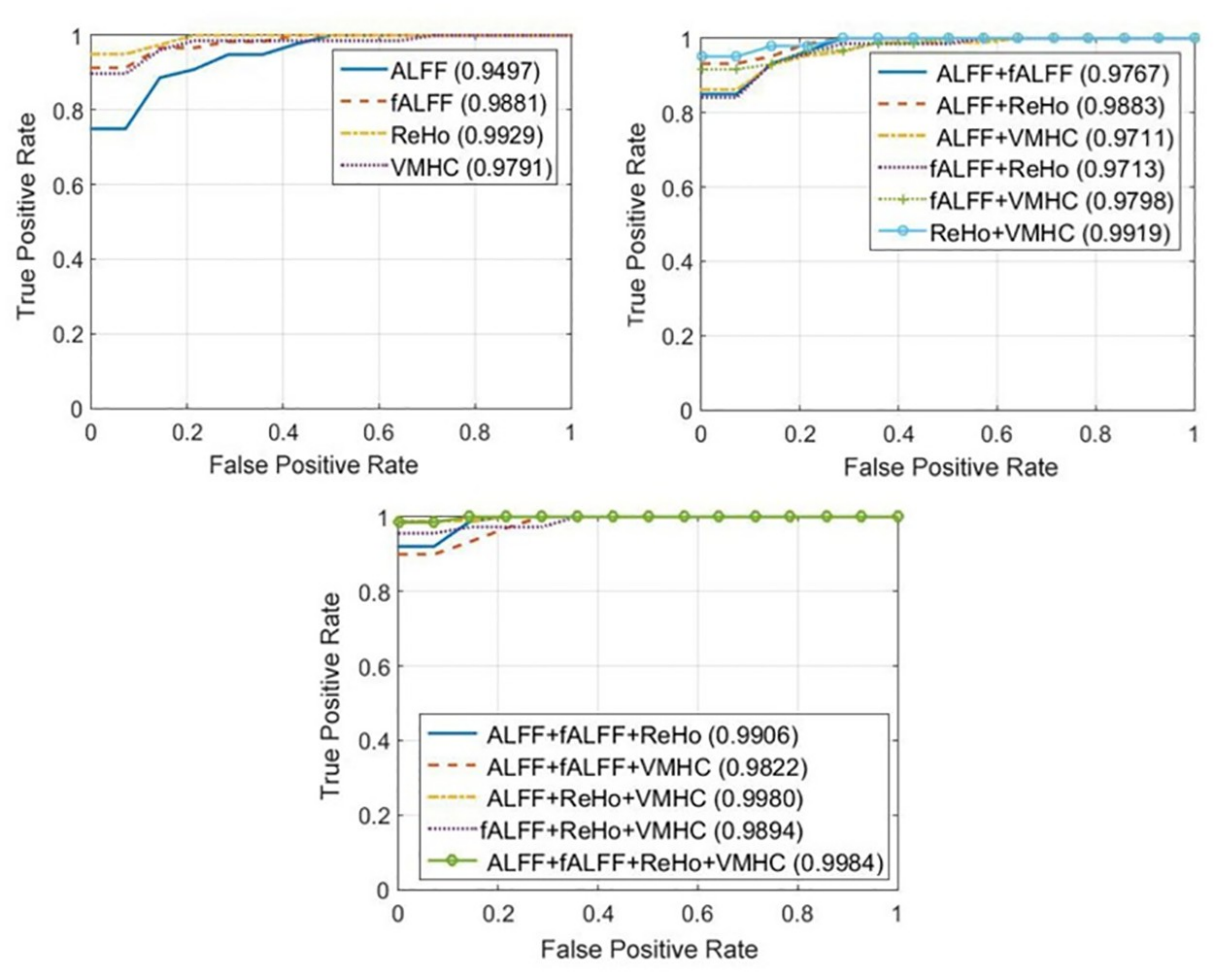
Localization of the areas in the AAL atlas. Circles represent AAL nodes. The blue circles represent the areas with higher discriminability between healthy and schizophrenia groups and the green circles represent unaffected areas.

The discriminative ALFF, fALFF, ReHo and VMHC connectivity maps were constructed using a two-sample t-test to statistically verify the significance of the difference between healthy subjects and schizophrenic patients. The significance level was set at the corrected p < 0.05 for multiple testing using the false discovery rate (FDR) method [82] (min z > 2.3, cluster significance: p < 0.05). The most discriminative features for classification are shown in Table 7. Moreover, these regions are highlighted using BrainNet Viewer [83] in Fig 4. The top features are listed in descending order of their weights. The highly activated regions include the lingual gyrus (LING), Precuneus (PCUN), anterior cingulate and paracingulate gyri (ACG), parahippocampal gyrus (PHG), precental gyrus (PreCG), cerebelum_6 (CRBL6), temporal pole: middle temporal gyrus (TPOmid), vermis_10 (VER10), middle frontal gyrus, orbital part (ORBmid), cerebelum_Crus2 (CERcr2), superior parietal gyrus (SPG), anterior cingulate and paracingulate gyri (ACG), cerebelum_7 (CER7) and postcentral gyrus (PoCG).

**Table 7:** The most discriminative measures and corresponding AAL regions.

Feature	Anatomical Location (AAL* areas)	Hemisphere	Peak MNI Coordinate			Peak Value
			X (mm)	Y (mm)	Z (mm)	
ReHo	Lingual gyrus	R	7	-46	5	3.078
	Precuneus	R	5	-46	7	3.078
	Precuneus	L	-9	-46	72	-2.318
	Anterior cingulate and paracingulate gyri	R	3	37	7	-1.949
ALFF	Parahippocampal gyrus	R	21	13	-29	2.402
	Precental gyrus	L	-16	-6	69	2.243
	Cerebelum_6	L	-37	-39	-31	-1.925
	Temporal pole: middle temporal gyrus	R	40	13	-43	-0.804
fALFF	Vermis_10	-	2	-41	-39	2.597
	Middle frontal gyrus, orbital part	L	-14	71	-4	2.291
	Cerebelum_Crus2	R	17	-82	-47	-1.679
	Superior parietal gyrus	R	17	-62	64	-1.643
VMHC	Parahippocampal gyrus	L	-18	-24	-14	2.665
	Anterior cingulate and paracingulate gyri	L	2	37	7	1.949
	Cerebelum_7	R	18	-81	-49	-1.679
	Postcentral gyrus	L	-65	-19	31	-1.181

*Automated Anatomical Labeling (90 Regions)

In comparison to healthy controls, the schizophrenic patients showed significant ReHo increases in the right LING and the right PCUN. Also, ALFF increases in the right PHG and the left PreCG, while fALFF increases in VER10 and the left ORBmid. As will, VMHC increases in the left PHG and the left ACG. Schizophrenic patients showed significant ReHo decreases in the left PCUN and the right ACG. Also, ALFF decreases in the right CERcr2 and the right TPOmid, while fALFF decreases in the right CERcr2 and the right SPG. In addition, VMHC decreases in the right CER7 and the left PoCG.

### 3.5 Robustness to noise

**Fig 5:**
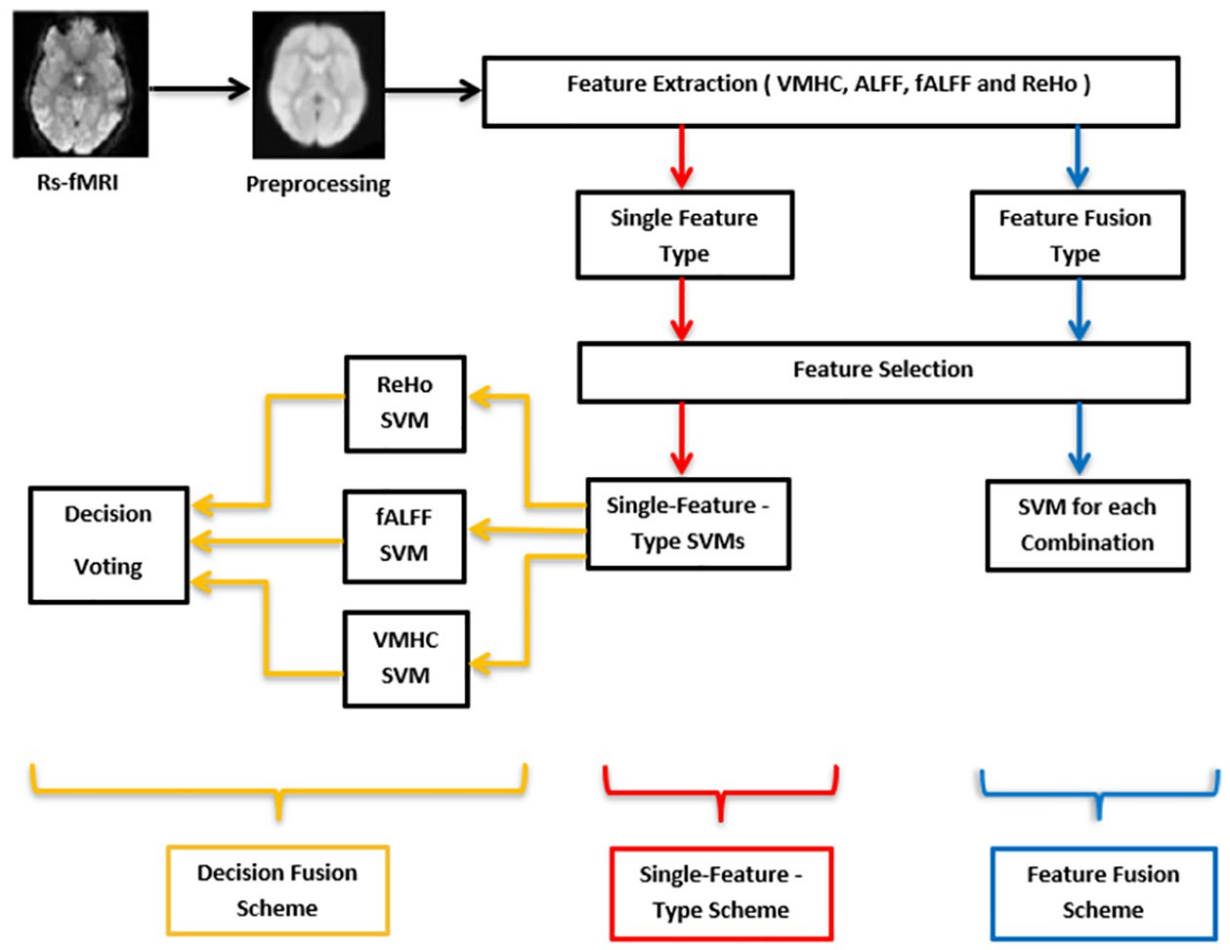
Schizophrenia detection performance outcomes under the Rician noise conditions for the best classifiers with single, pairwise, triple, and quadruple feature combinations as well as the decision-fusion classifier.

We performed some additional experiments to investigate the robustness of the proposed method with different feature combinations. In particular, we added Rician noise with two different levels of *σ* = 1 and *σ* = 2 to the test data. The performance outcomes under these noise conditions are summarized in Tables 8 and 9 for the single-feature-type classifiers and fused-feature classifiers, respectively. Moreover, the performance outcomes under the noise conditions are visualized for the best classifiers with single, pairwise, triple, and quadruple feature combinations as well as decision-fusion classifier in Fig 5.

**Table 8:** Effects of Rician noise on schizophrenia detection performance with single-feature-type and decision-level fusion schemes for fMRI data contaminated with Rician noise levels of *σ* = 1 and *σ* = 2.

Feature type	Sensitivity (%)			Specificity (%)			Accuracy (%)		
	Noiseless	*σ* = 1	*σ* = 2	Noiseless	*σ* = 1	*σ* = 2	Noiseless	*σ* = 1	*σ* = 2
ALFF	81.33	79.89	78.36	**99.50**	**97.23**	83.89	90.71	87.95	83.36
fALFF	91.56	89.43	87.78	93.36	91.32	82.57	92.71	89.86	84.85
VMHC	**94.25**	**92.64**	85.26	93.89	92.43	84.35	93.85	90.63	85.22
ReHo	94.08	92.33	**88.26**	95.78	93.66	**86.62**	**94.57**	**92.46**	**86.46**
Decision-fusion classifier	**98.33**	**96.43**	**88.42**	**96.83**	**94.53**	**86.65**	**97.85**	**95.78**	**88.54**

**Table 9:** Effects of Rician noise on schizophrenia detection performance with feature-level fusion of different pairwise, triple, and quadruple combinations of single feature types for fMRI data contaminated with Rician noise levels of *σ* = 1 and *σ* = 2.

	Feature type	Sensitivity (%)			Specificity (%)			Accuracy (%)		
		Noiseless	*σ* = 1	*σ* = 2	Noiseless	*σ* = 1	*σ* = 2	Noiseless	*σ* = 1	*σ* = 2
Pairwise features	ALFF+fALFF	**98.80**	**96.32**	**86.85**	**96.32**	94.82	**88.57**	**97.71**	94.67	**87.71**
	ALFF+ReHo	97.40	94.83	85.77	97.64	95.54	86.40	97.42	95.52	86.57
	ALFF+VMHC	96.93	93.45	84.21	96.86	93.27	83.85	96.71	94.55	85.42
	fALFF+ReHo	95.08	93.91	85.85	98.51	**96.54**	86.57	97.42	95.62	86.61
	fALFF+VMHC	96.85	94.32	84.46	98.00	96.08	83.86	97.57	95.33	84.93
	ReHo+VMHC	95.61	92.66	83.80	**99.77**	96.54	84.77	97.57	**95.86**	85.71
Triple features	ALFF+fALFF+ReHo	**98.62**	**96.64**	84.66	98.62	96.53	85.51	**97.85**	95.41	86.85
	ALFF+fALFF+VMHC	97.21	95.34	**86.08**	98.72	**96.74**	**85.72**	97.85	**95.92**	**87.93**
	ALFF+ReHo+VMHC	92.46	89.45	83.40	95.26	92.34	82.86	93.85	91.68	83.42
	fALFF+ReHo+VMHC	95.22	92.46	84.21	97.35	94.23	83.22	95.71	92.55	84.64
Quadruple features	ALFF+fALFF+ReHo+VMHC	**99.71**	**97.64**	**89.62**	**97.66**	**96.32**	**88.93**	**98.71**	**96.21**	**90.71**

For the best single-feature-type classifier, namely the ReHo-based classifier, the 95.57% detection accuracy dropped by 2.11% and 6.11% with the two noise levels, respectively. For the decision-level fusion classifier, the 97.85% detection accuracy dropped by 2.07% and 9.31% with the two noise levels, respectively. The best pairwise-feature-type classifier, i.e. the classifier based on the ALFF and fALFF features, the 97.71% detection accuracy dropped by 1.85% and 10% with the two noise levels, respectively. For the best triple-feature-type classifier, that is the one based on the ALFF-fALFF-VMHC feature combination, the 97.85% detection accuracy dropped by 2.13% and 9.92% with the two noise levels, respectively. Finally, for the classifier with the quadruple feature combination, the 98.71% detection accuracy dropped by 2.50% and 8% with the two noise levels, respectively. These results show that all classifiers essentially experience similar degradation rates under the Rician noise conditions. While the degradation rate is small for a noise level of *σ* = 1, this rate becomes understandably severe for the high noise level of *σ* = 2. Nevertheless, the classifier with the quadruple combination shows better robustness with the the highest accuracy under the noise-free condition and the smallest performance drop (8%) under the high noise condition *σ* = 2. Similar degradation patterns can be observed for the other performance metrics.

## 4 Discussion

In this paper, we investigated different fusion schemes of resting-state functional activity measures for discriminating between schizophrenic and healthy subjects. The improvements in classification performance can be ascribed to the choice of the feature selection and fusion approaches. For the decision fusion scheme in Table 3, the results outperformed those of the single-feature-type schemes. This shows that the fusion of the decisions of weak classifiers leads to more accurate classification performance [84]. Further, the feature fusion schemes in Table 4 show even better performance enhancements. This additional improvement can be ascribed to the fact that the feature-fusion schemes combine and optimize the selection of features, while the decision-level fusion scheme classifies samples by merely conducting a vote among only three single-feature-type classifiers.

**Fig 6:**
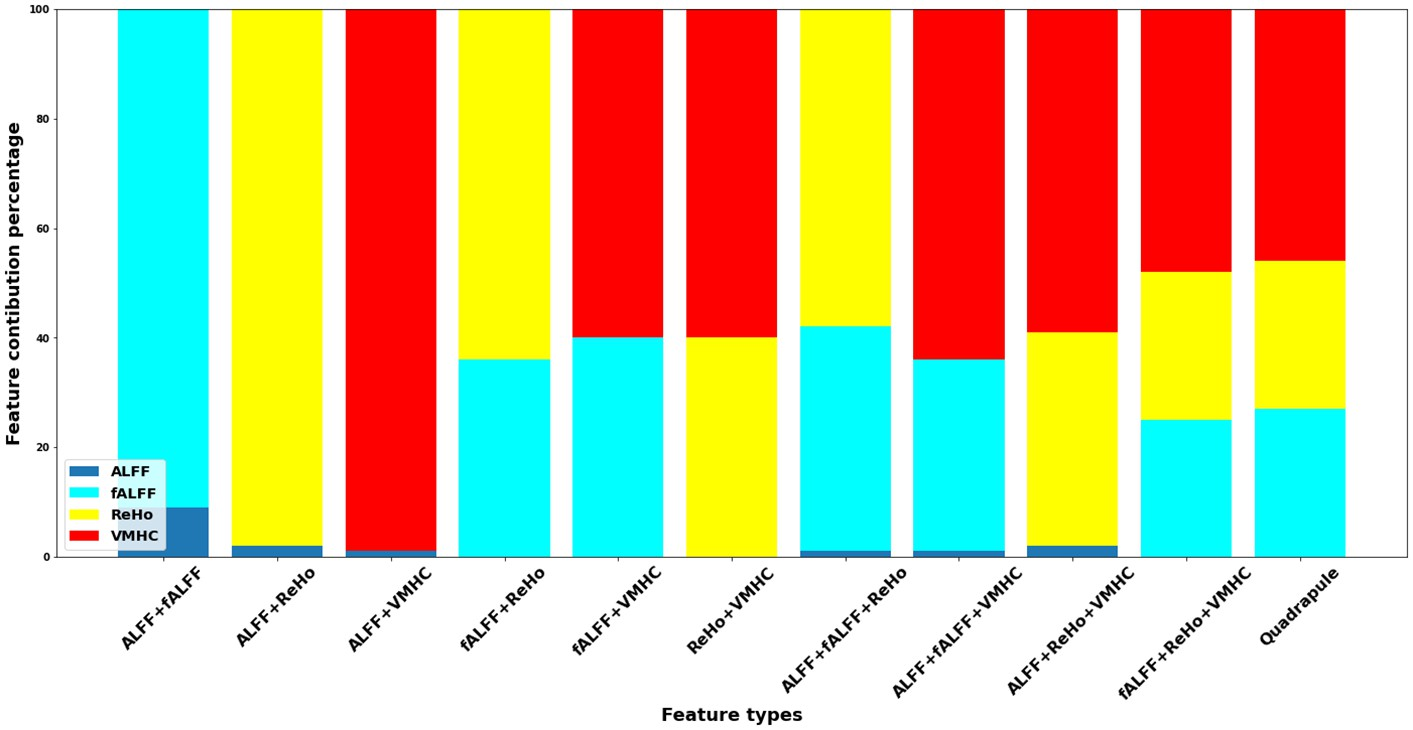
Contribution percentage of each of the ALFF, fALFF, ReHo, VMHC feature types in the performance of the classifiers with pairwise, triple, and quadruple feature combinations.

We can understand the improvements obtained by different variants of the feature fusion classifiers by looking at the contributions of the single feature types to each of these classifiers. Specifically, Fig 6 shows the selected percentages of each of the ALFF, fALFF, ReHo, and VMHC feature types for optimizing the performance of the classifiers with pairwise, triple, and quadruple feature combinations. Obviously, each combination has different selections of individual feature types. For example, for pairwise combinations, the ALFF feature type is dominated by the other features; the fALFF type is dominated by the ReHo and VMHC ones; and the ReHo type is slightly dominated by the VMHC one. For triple combinations, the ALFF type consistently shows zero or marginal contributions, while the fALFF features are generally dominated by the ReHo and VMHC features. For the quadruple feature combination associated with the best performance, the same pattern is observed where the ALFF type has no contribution, while the VMHC type has the highest contribution followed by the fALFF and ReHo types.

To put our results in context with other relevant studies, we examined the schizophrenia detection results in some of these studies. Firstly, the high performance metrics of our method agree with the high accuracies reported by other studies on the COBRE dataset. For example, Qureshi et al. [85] achieved an accuracy of 100% on the COBRE dataset with 10-fold cross-validation scheme and extreme learning machines (ELM). Also, Chyzhyk et al. [86] attained a 100% accuracy in the classification of healthy subjects and schizophrenic patients with and without auditory hallucinations. Juneja et al. [87] obtained a 98% accuracy on a multisite dataset from the Function Biomedical Informatics Research Network (FBIRN). Other schizophrenia detection methods achieved lower accuracies ranging from 62% to 94% for different variations of the schizophrenia classes, the image modalities and the collected datasets [88].

Furthermore, the performance metrics of the schizophrenia detection systems rank differently compared to other brain diseases [88]. On the one hand, the inter-quartile range of the reported overall accuracies for schizophrenia (76%-92%) is generally higher than those associated with stroke (67%-87%), ADHD (67%-76%) and depression (77%-86%). On the other hand, the inter-quartile range for schizophrenia is generally lower than those of the Alzheimer’s disease (AD) (86%-97%) and Parkinson’s disease (PD) (87%-98%).

The vertical averaging method was employed to plot the ROC curves as shown in Fig 3. We noticed that the AUC values of the combinations of features are slightly better than those based on single feature types.

The significant regions that are shown in Table 7 are in agreement with the previous findings. Studies based on ReHo features reported an increase in ReHo values in the right LING, the right PCUN and a decrease in ReHo values in the left PCUN and the right ACG [89, 90]. ALFF-based studies reported significant ALFF increases in the right PHG and decreased ALFF values in the right PHG and the left PreCG [91]. Also, fALFF-based studies demonstrated significant fALFF increases in VER10 and the left ORBmid and decreased fALFF values in the right CERcr2 and the right SPG [91, 92]. Finally, VMHC-based studies reported significant VMHC increases in the left PHG and the left ACG and decreased VMHC values in the right CER7 and the left PoCG [93]. As shown in Table 3, the standard deviation of the accuracy results for the outer 10-fold cross-validation scheme is considered small, and this demonstrates the low bias and high robustness of the proposed system for schizophrenia diagnosis. Moreover, the extracted features can be employed as biological markers that may help identify subjects at increased risk of disease development, and hence improve disease prognosis. Furthermore, the localization of the affected regions, in Table 7, can be employed in further research to localize and understand how the different regions are affected and changed through the progression of the disease.

## 5 Conclusion

In this paper, we introduced different feature-level and decision-level fusion schemes for discriminating between schizophrenic and healthy subjects and identifying schizophrenia-affected brain regions using whole-brain Rs-fMRI analysis. The highest average test accuracy of 98.71% was obtained with feature fusion of the four types of features considered in this paper. In summary, our work employs optimized feature selection algorithms, explores different fusion schemes, and exploits a large-scale dataset for schizophrenia detection. For future work, we seek to address this detection problem with deep learning and graph-theoretic techniques for two main reasons. Firstly, in practical applications for schizophrenia detection, the actual test data may be contaminated by noise and artifacts. Under these conditions, the handcrafted features may be not quite robust. This is why other graph-theoretic or deep learning methods with better noise robustness measures should be sought. Secondly, although the employed COBRE dataset is considered big compared to the other available datasets, a larger dataset is still needed with few hundreds of MRI volumes, acquired from different centers, with different MRI machines, and at different specifications. With these large real-world data variations, our current handcrafted-feature method may not achieve the same performance outcomes, and hence deep learning architectures would be needed to effectively capture the increasing data complexity.
